# Artificial Intelligence in Medicine: Cross-Sectional Study Among Medical Students on Application, Education, and Ethical Aspects

**DOI:** 10.2196/51247

**Published:** 2024-01-05

**Authors:** Lukas Weidener, Michael Fischer

**Affiliations:** 1 Research Unit for Quality and Ethics in Health Care UMIT TIROL – Private University for Health Sciences and Health Technology Hall in Tirol Austria

**Keywords:** artificial intelligence, AI technology, medicine, medical education, medical curriculum, medical school, AI ethics, ethics

## Abstract

**Background:**

The use of artificial intelligence (AI) in medicine not only directly impacts the medical profession but is also increasingly associated with various potential ethical aspects. In addition, the expanding use of AI and AI-based applications such as ChatGPT demands a corresponding shift in medical education to adequately prepare future practitioners for the effective use of these tools and address the associated ethical challenges they present.

**Objective:**

This study aims to explore how medical students from Germany, Austria, and Switzerland perceive the use of AI in medicine and the teaching of AI and AI ethics in medical education in accordance with their use of AI-based chat applications, such as ChatGPT.

**Methods:**

This cross-sectional study, conducted from June 15 to July 15, 2023, surveyed medical students across Germany, Austria, and Switzerland using a web-based survey. This study aimed to assess students’ perceptions of AI in medicine and the integration of AI and AI ethics into medical education. The survey, which included 53 items across 6 sections, was developed and pretested. Data analysis used descriptive statistics (median, mode, IQR, total number, and percentages) and either the chi-square or Mann-Whitney *U* tests, as appropriate.

**Results:**

Surveying 487 medical students across Germany, Austria, and Switzerland revealed limited formal education on AI or AI ethics within medical curricula, although 38.8% (189/487) had prior experience with AI-based chat applications, such as ChatGPT. Despite varied prior exposures, 71.7% (349/487) anticipated a positive impact of AI on medicine. There was widespread consensus (385/487, 74.9%) on the need for AI and AI ethics instruction in medical education, although the current offerings were deemed inadequate. Regarding the AI ethics education content, all proposed topics were rated as highly relevant.

**Conclusions:**

This study revealed a pronounced discrepancy between the use of AI-based (chat) applications, such as ChatGPT, among medical students in Germany, Austria, and Switzerland and the teaching of AI in medical education. To adequately prepare future medical professionals, there is an urgent need to integrate the teaching of AI and AI ethics into the medical curricula.

## Introduction

### Background

Artificial intelligence (AI) has attracted both public and scientific interest and is amplified by the emergence and greater accessibility of chat-based applications such as ChatGPT (OpenAI, LLC) and Bard (Google, LLC). For several years, the medical field has been an active and expanding area of research on the application of AI [1]. As of now, AI is used in diverse medical specializations, including dermatology, radiology, and pathology [2-4].

Although the history of AI can be traced back to the 1950s, the public’s unrestricted access to highly advanced large language models, such as ChatGPT, can be seen as a significant turning point in the history of AI [5,6]. Early studies demonstrated that ChatGPT is capable of successfully completing the written portion of the United States Medical Licensing Examination [7]. Given the capabilities of AI-based chat applications such as ChatGPT in medicine, further studies have highlighted their potential use in providing information on cancer, assisting in clinical diagnoses, authoring scientific research articles, and patient communication [8-10]. Considering the wide availability and integration of medical knowledge in this application, its increasing use in medicine and among medical students is foreseeable [11].

Despite the long history of AI and the increasing adoption of this technology, there is disagreement regarding its definition among the scientific community [12]. There is a consensus within the scientific community on distinguishing between the so-called strong AI, also known as “artificial general intelligence,” and weak AI or “artificial narrow intelligence” [13]. This categorization is based on the capabilities of AI or its areas of application [13]. Strong AI, recognized for its human-equivalent intellectual abilities and knowledge, stands in contrast to weak AI, which refers to AI solutions capable of accomplishing specific tasks effectively [13]. The area of weak AI can be further divided into the so-called statistical AI and symbolic AI. The field of statistical AI also includes machine learning and deep learning, on which large language models such as ChatGPT are based [13]. Areas of application for symbolic AI in medicine include expert systems (eg, clinical decision support systems), which make decisions based on explicit knowledge in the form of predefined rules [14].

Considering the likely significant impact the implementation and use of AI in medicine is poised to make, a growing body of literature advocates the inclusion of AI-related content in medical curricula [15-18]. In addition to implications for the medical profession and patient care, medical students are expected to face new ethical challenges posed by the use of AI in medicine [15,19]. Despite the potentially significant ethical challenges anticipated from the deployment of AI in medicine, such as the possibility of discrimination due to biases in the data used for training or effects on patient autonomy, there is a near-complete absence of scientific publications on specific teaching content or methods related to AI ethics as part of medical higher education.

In addition to the lack of specificity regarding teaching content on AI and AI ethics, the absence of studies on medical students’ perception of AI ethics education (including teaching content) is notable [20,21]. It is essential to point out that the current state of research regarding medical students’ perceptions and assessments of AI application in medicine largely represents a knowledge base that predates the advent of large language models such as ChatGPT. With the ubiquity of the aforementioned AI applications at the time of this publication, it is reasonable to expect that medical students’ assessments of AI implementation in medicine will deviate significantly from earlier publications within this area of research, highlighting the need for further research.

### Objective

This study aimed to explore how medical students perceive the use of AI in medicine, as well as the teaching of AI and AI ethics (including prospective AI ethics teaching topics). In this context, the introduction and accessibility of large language models such as ChatGPT should be emphasized, leading to the following research question: how do medical students from Germany, Austria, and Switzerland perceive (1) the application of AI in medical practice, (2) the integration of AI and AI ethics into medical education, and (3) AI ethics teaching content in their curriculum in accordance with the use of AI-based chat applications such as ChatGPT?

To address this research question, the participating medical students were divided into 2 groups based on their prior use of AI-based (chat) applications, such as ChatGPT.

## Methods

### Overview

This cross-sectional study was conducted between June 15 and July 15, 2023. During this time frame, an invitation to participate in the study was sent to medical students who were regularly enrolled in universities in Germany, Austria, and Switzerland. The study sample included medical students from all academic semesters, including those in practically oriented semesters such as the practical year in Germany. Participation in the study was voluntary and there were no consequences for nonparticipation. The study used an anonymous web-based survey, with recruitment facilitated through email invitations and assistance from various medical student associations, unions, and councils in their respective countries. To minimize potential selection bias, the survey invited medical students from various universities and academic semesters in Germany, Austria, and Switzerland. This strategy ensured a broad and representative sample of the participants. Moreover, careful construction and pretesting of the survey were conducted to minimize potential response biases. Before the official data collection, a pretest was conducted with 11 medical students from the target population. The web-based survey provider, “LimeSurvey” was used for both the pretest and the main study.

### Ethical Considerations

The Research Committee for Scientific Ethical Questions granted ethical approval for this study (3181) on January 16, 2023.

### Survey Development

The survey used for data collection was developed based on existing scientific publications [15,22]. Owing to the lack of references in the areas of AI teaching, AI ethics, and recent developments in AI, most items used for the survey were newly formulated. The survey comprises 53 items, including both questions and statements. During the development process, these items were distributed across 6 parts, with some contingent on the responses to the preceding items. The first part aimed to collect information on the demographic characteristics and educational background of the participants. To address the research question of this study, participants were divided into 2 groups based on their responses to questions related to their prior use of AI-based (chat) applications such as ChatGPT. The second part sought to gather information about the students’ previous experiences with AI-based (chat) applications. In the third part, the students were asked to rate various statements regarding the use of AI in medicine. The fourth and fifth parts aimed to capture students’ evaluations of statements about AI teaching and ethics, respectively. The sixth part assessed the perceived relevance of the potential teaching content to AI ethics. The items in parts 3 to 6 were evaluated using a 5-point Likert scale. Before the survey was conducted, 2 experts in ethics and AI evaluated the survey and their recommendations were incorporated. Upon receiving expert feedback, the teaching topic of “data privacy” was introduced as a distinct subject under AI ethics. Previously, this was encompassed within the broader “safety” category. Furthermore, to enhance clarity, the term “knowingly” was incorporated into Q12. This adjustment acknowledges that the application of AI in medicine may not always be transparent.

### Survey Pretest

To assess the comprehensibility and relevance of the survey, a pretest was conducted with 11 medical students, who subsequently provided feedback. This feedback led to 6 relevant modifications aimed at enhancing clarity, relevance, and user-friendliness. Because of the feedback provided, questions Q1 through Q4 and Q6 were specified by adding examples following each question. The changes made to the questions are highlighted in italics:

1. Q1. Have you already received education in the field of ethics within your regular medical studies? *(eg, as part of the History, Ethics, and Theory of Medicine course)*
2. Q2. Have you already received education in the field of AI in your regular medical studies? *(eg, as part of medical statistics or informatics)*
3. Q3. Have you already received education in the field of AI outside of your regular medical studies? *(eg, in the form of further training, own research)*
4. Q4. Have you already received education in the field of AI ethics within your regular medical studies? *(eg, as part of the History, Ethics, and Theory of Medicine course)*
5. Q6. Have you already received instruction in the field of AI ethics outside of your regular medical studies? *(eg, in the form of further training, own research)*

Similarly, statement 27 (S27) was further improved by adding examples from various fields to underscore the multidisciplinary context: “AI ethics should be taught by experts from various fields *(eg, medicine, computer science, philosophy)* to ensure a multidisciplinary perspective on AI ethics.”

To improve the survey’s user experience, conditional logic was integrated so that questions Q5 and Q7 appeared only in response to the specific preceding answers. Both question Q5 and question Q7 were designed to explore the specific content covered in AI ethics education. These questions were identical in wording: “Which of the following contents were covered as part of the instruction/education?” Question Q5 was presented exclusively to participants who answered “yes” to question 4, which focused on AI ethics education within their regular medical studies. Similarly, question Q7 was shown only to those who responded “yes” to question 6, focusing on AI ethics education outside of their regular medical curriculum. This strategic modification not only streamlined the survey’s presentation but also minimized the immediate visual content, reducing complexity.

### Sample Size Calculation

The sample size for this study was calculated before data collection using Cochran sample size formula (n = [Z^2 * p * (1-p)] / E^2) [23]. The total population size used for the calculation, which represents the number of medical students enrolled at the end of the winter semester in 2022, was 130,601 across the 3 countries included in the study. This figure includes 105,275 medical students from Germany (accounting for 80.61% of the total), 17,826 from Austria (13.65%), and 7500 from Switzerland (5.74%) [24-26]. This summation was performed based on the primary research question and was predicated on the assumption that the prevalence of AI-based (chat) applications, such as ChatGPT, among medical students does not vary significantly across these countries. A confidence level of 95% (*Z*=1.96) and a margin of error of 5% were used to determine the sample size. The proportion (p) was derived from a pretest involving a separate group of 11 medical students of which 5 were already using large language models such as ChatGPT before the study (*P*=.45). Cochran’s formula yielded a sample size of 380 medical students. As the study was conducted using a web-based survey with recruitment via email, an estimated dropout rate of 40% was factored in. To achieve a calculated sample size of 380 participants, at least 532 students were targeted during the recruitment process. To ensure adequate representation based on the proportion of medical students within each country of interest, the study aimed to include at least 306 medical students from Germany, 52 from Austria, and 22 from Switzerland in the data collection and analysis process. Note that these are rounded values given that the actual calculations result in noninteger numbers.

### Data Analysis

Collected data were evaluated using SPSS (version 28; IBM Corp), LimeSurvey (LimeSurvey GmbH), and Microsoft Excel (version 16.73). Descriptive statistics were calculated for all survey variables, including the median, IQR, mode, total number, and percentages. For further statistical analysis, the chi-square test of independence was used to compare the 3 groups. When significant differences were observed in the chi-square test, post hoc analysis was performed using the adjusted residuals method to specify which specific groups or categories contributed to the observed significance. In addition, *z* scores were calculated to facilitate the comparison of responses across different groups. These were computed using the 2-sided test formula z = (X – μ) / σ, where X represents the value of the response, μ is the mean of the responses for the group, and σ is the SD within that group. The calculation of *z* scores enabled the quantification of the deviation of each response from the group mean in terms of SDs. The Mann-Whitney *U* test was used for the statistical comparison of 2 independent groups; for further statistical analysis, the chi-square test of independence was used to compare the 3 groups, and the Mann-Whitney *U* test was used for the statistical comparison of 2 independent groups. For statistical analysis, the responses to the Likert scale were recoded into a numerical format (“I strongly disagree”=1, “I disagree”=2, “undecided”=3, “I agree”=4, “I strongly agree =5). For all statistical tests performed, the significance level was set at α=.05, and a value of *P*≤.05 was considered statistically significant. Only complete data sets were included in the data analysis to avoid potential biases that could arise from replacing or estimating the missing values (list-wise deletion).

## Results

### Overview

In total, 521 medical students participated in the survey, yielding 487 complete and valid data sets for the statistical analysis. The survey invitations were disseminated via email with the help of medical student associations, unions, and councils. The total number of medical students reached and the precise response rate could only be approximated. On the basis of the feedback received from the engaged medical student councils, we estimated that at least 2000 medical students were approached. This would be equal to a response rate of 24.35% (487/2000). Our sample size calculation was based on the assumption that the use of AI-based (chat) applications such as ChatGPT does not diverge markedly among medical students from each of the countries of interest, namely Germany, Austria, and Switzerland. Consequently, the chi-square test of independence was used for statistical evaluation. We posited a null hypothesis (H₀) asserting no association between the variables (use of AI-based applications and country of study) and an alternative hypothesis (H₁) suggesting an association between these variables. The chi-square test returned a value of *P*=.96, which exceeded the predetermined level of significance. As such, we did not reject the null hypothesis, leading us to conclude that there is no statistically significant association between the use of AI-based (chat) applications and country of study among the surveyed medical students, given that each individual fits into one category for each variable.

### Part 1: Demographics and Educational Background

Of the medical students who participated in the survey, the majority were women (270/487, 55.4%). The largest demographic age was between 20 and 25 years (301/487, 61.8%), and most students were enrolled in Germany (296/487, 60.7%). The German contingent of respondents was slightly below our target size of 306, representing a 3.3% (296/306) shortfall. However, participation from Austria exceeded our initial target of 52 students by a substantial margin, with 105 respondents indicating enrollment in Austria, denoting an overachievement rate of 202% (105/52). Similarly, Swiss representation surpassed our initial target of 22 students, with 86 respondents registered in Switzerland, marking an overachievement of 391% (86/22). Most of the surveyed students were in the clinical stage (CS) of their study (277/487, 56.9%), followed by those in their practical years (63/487, 12.9%). Comprehensive demographic characteristics are presented in Table 1.

The respondents were also asked about their educational backgrounds in ethics, AI, and AI ethics. Most participants (425/487, 87.2%) reported having received ethics education. However, a considerably smaller proportion of respondents claimed that they had received prior education in AI as part of their medical curriculum (26/487, 5.3%), with an additional 10.5% (51/487) having obtained such knowledge outside of their regular medical studies. Few participants had been exposed to AI ethics education within their medical curriculum (21/487, 4.3%), with a small number reporting having learned about AI ethics outside their regular curriculum (51/487, 6.8%). The most common subjects covered in AI ethics education were bias (15/487, 3.1% within and 14/487, 2.9% outside regular studies) and explainability (12/487, 2.5% within and 20/487, 4.1% outside regular studies). Detailed responses related to the participants’ educational background are shown in Table 2.

**Table 1:** Demographic characteristics of medical students (n=487).

Characteristics		Medical students, n (%)
**Gender**		
	Woman	270 (55.4)
	Man	203 (41.7)
	Nonbinary	3 (0.6)
	Prefer not to say	11 (2.3)
**Age (y)**		
	<20	56 (11.5)
	20-25	301 (61.8)
	26-30	92 (18.9)
	31-35	28 (5.7)
	>35	10 (2.0)
**Country of enrollment (medical studies)**		
	Germany	296 (60.7)
	Austria	105 (21.5)
	Switzerland	86 (17.7)
**Stage of study**		
	Preclinical	57 (11.7)
	Clinical	277 (56.9)
	Practical year	63 (12.9)
	Elective year	26 (5.3)
	Bachelor	46 (9.4)
	Master	18 (3.7)

**Table 2:** Educational background of the participating medical students from Germany, Austria, and Switzerland (n=487).

Question		Participants, n (%)
**Q1: Have you already received education in the field of ethics *within* your regular medical studies? (eg, as part of the History, Ethics, and Theory of Medicine course)**		
	Yes	425 (87.2)
	No	62 (12.7)
**Q2: Have you already received education in the field of artificial intelligence *within* your regular medical studies? (eg, as part of medical statistics or informatics)**		
	Yes	26 (5.3)
	No	461 (94.7)
**Q3: Have you already received education in the field of artificial intelligence *outside of* your regular medical studies? (eg, in the form of further training, own research)**		
	Yes	51 (10.5)
	No	436 (89.2)
**Q4: Have you already received education in the field of artificial intelligence ethics *within* your regular medical studies? (eg, as part of the History, Ethics, and Theory of Medicine course)**		
	Yes	21 (4.3)
	No	466 (95.7)
**Q5: Which of the following contents were covered as part of the education?^a,b^**		
	Informed consent	11 (2.3)
	Bias	15 (3.1)
	Data privacy	13 (2.7)
	Explainability	12 (2.5)
	Safety (of AI-based applications)	10 (2)
	Fairness	5 (1)
	Autonomy	8 (1.6)
	Responsibility	8 (1.6)
**Q6: Have you already received education in the field of artificial intelligence ethics *outside of* your regular medical studies? (eg, in the form of further training, own research)**		
	Yes	33 (6.8)
	No	454 (93.2)
**Q7: Which of the following contents were covered as part of the education?^b,c^**		
	Informed consent	10 (2)
	Bias	14 (2.9)
	Data privacy	17 (3.5)
	Explainability	20 (4.1)
	Safety (of artificial intelligence-based applications)	18 (3.7)
	Fairness	12 (2.5)
	Autonomy	14 (2.9)
	Responsibility	19 (3.9)

^a^Question 5 was exclusively displayed to participants who responded to question 4 with “yes.”

^b^An explanation of the contents of Q5 and Q7 is provided in the text.

^c^Question 7 was exclusively displayed to participants who responded to question 6 with “yes.”

### Part 2: Use of AI-Based (Chat) Applications

With regard to the use of AI-based (chat) applications such as ChatGPT (OpenAI), Bard (Google), Bing Chat (Microsoft Inc), and Jasper Chat (Jasper AI, Inc), 38.8% (189/487) of the respondents reported prior use of these platforms. Conversely, the vast majority (438/487, 89.9%) indicated that they did not knowingly use other AI-based medical applications. Of the 298 respondents who had not previously used an AI-based chat application, 76.9% (n=229) expressed an interest in future use. Among the respondents who reported prior use of AI-based (chat) applications, nearly half had used such an application for 1-3 hours over the past week (91/189, 48.2%). Of this group, 73% (138/189) indicated using an AI-based (chat) application in a medical context, with the most common use being querying medical knowledge (74/138, 53.6%). The results of this survey are summarized in Table 3.

**Table 3:** Answers to the use of AI^a^-based (chat) applications of participants (n=487).

Question		Participants, n (%)
**Q8: Have you already used an AI-based (chat) application such as ChatGPT (OpenAI), Bard (Google), Bing chat, or Jasper Chat?**		
	Yes	189 (38.8)
	No	298 (61.2)
**Q9: Have you knowingly ever used AI-based medical applications, such as image-based diagnostic tools in radiology?**		
	Yes	49 (10.1)
	No	438 (89.9)
**Q10: Are you interested in using an AI application as part of your medical studies in the future?^b^; n=298**		
	Yes	229 (76.9)
	No	69 (23.1)
**Q1: Approximately how many hours have you used the AI-based (chat) application in the last week (7 d)^c^; (n=189)**		
	<1 h	73 (38.6)
	1-3 h	91 (48.2)
	4-6 h	19 (10)
	7-9 h	3 (1.6)
	10-12 h	2 (1.1)
	>12 h	1 (0.5)
**Q12: Have you already used the AI-based (chat) application in a medical context? (eg, for explaining medical conditions or medical questions)^d^; (n=189)**		
	Yes	138 (73)
	No	51 (26.7)
**Q13: For which of the following objectives have you already used the AI-based (chat) application in the medical context?^e^; (n=138)**		
	Therapy suggestions	18 (13)
	Querying medical knowledge	74 (53.6)
	Diagnostic support	5 (3.6)
	Explanation of pathologies	41 (29.7)

^a^AI: artificial intelligence.

^b^Question 10 was exclusively displayed to participants who responded to questions 8 and 9 with “no.”

^c^Question 11 was exclusively displayed to participants who responded to question 8 with “yes.”

^d^Question 12 was exclusively displayed to participants who responded to question 8 with “yes.”

^e^Question 13 was exclusively displayed to participants who responded to question 12 with “yes.”

### Part 3: AI in Medicine

In the third part of the survey, participants’ attitudes toward the role of AI’s in medicine were examined. Of the 487 respondents, 71.7% (n=349) agreed or strongly agreed that the use of AI would bring about positive changes to medicine (S1). Similarly, 72.1% (350/487) believed that AI could find practical applications in medicine (S2). When comparing the responses between those who had used AI-based applications and those who did not, significant differences were identified for each statement using the Mann-Whitney *U* test (S1: *P*=.003; S2: *P*=.002). Although both groups shared the same median and mode responses, their *z* scores suggested variations in their agreement levels. Specifically, respondents who had not previously used AI-based chat applications displayed a higher level of agreement with the statement in S1 (*z* score: −2.991). Conversely, those who had used AI-based applications exhibited greater concurrence with the statement in S2 (*z* score: 3.105).

When comparing the responses of those who had used AI-based chat applications and those who had not, no significant difference was observed regarding the subsequent 2 statements, S3 and S4, which were related to the influence on the choice of medical specialization and the potential reduction of jobs for medical staff. However, marked differences were identified when comparing the responses to statements S5 to S7 concerning improvements in patient care quality (S5: *P*<.001), diagnostic processes (S6: *P*=.002), and therapy selection (S7: *P*<.001). Although the overall agreement (either “agree” or “strongly agree”) was high for these statements (S5: 71%; S6: 76.4%; S7: 77.9%), *z* scores indicated greater agreement within the subgroup that had previously used AI-based (chat) applications (S5: *z* score=3.570; S6: *z* score=3.089; S7: *z* score=3.865).

No significant difference was found for statements S8 to S11 between the 2 groups, with comparable levels of overall agreement (“agree” or “strongly agree”) for each statement (S8: 31.8%; S9: 29.6%; S10: 25.9%; S11: 31.8%). However, a significant difference was observed for statement S12 (*P*=.02), with 95.3% of all respondents agreeing or strongly agreeing that the use of AI in medicine presents new ethical challenges. The *z* score (2.302), median (5), and mode (5) suggested a higher level of agreement among the groups that had previously used AI-based (chat) applications, such as ChatGPT. A statistical analysis of the third part of the survey is presented in Table 4. A detailed illustration of the perceptions of the surveyed medical students regarding the use of AI in medicine is provided in Table S1 in Multimedia Appendix 1.

**Table 4:** Statistical analysis of the perceptions of medical students regarding the use of artificial intelligence (AI)–based (chat) applications such as ChatGPT (OpenAI), Bard (Google), Bing Chat (Microsoft Inc), and Jasper Chat (Jasper AI, Inc) in medicine (n=487).

Statement and subgroup				Scores, median (IQR)		Scores, mode		*P* value			*Z* score	
The use of AI in medicine will...												
	**S1: ...positively change medicine**								.003			−2.990
		Subgroup 1: previous use of AI	4 (3.75-4.25)		4							
		Subgroup 2: no previous use of AI	4 (3-4)		4							
	**S2: ...find useful applications in medicine**								.002			3.101
		Subgroup 1: previous use of AI	4 (3.5-4.5)		4							
		Subgroup 2: no previous use of AI	4 (3-4)		4							
	**S3: ...influence the choice of my medical specialization**								.52			−1.474
		Subgroup 1: previous use of AI	3 (2-4)		2							
		Subgroup 2: no previous use of AI	3 (2-4)		2							
	**S4: ...reduce the number of jobs for medical staff**								.09			−1.707
		Subgroup 1: previous use of AI	3 (3-5)		4							
		Subgroup 2: no previous use of AI	3 (2-4)		2							
	**S5: ...improve the quality of patient care**								<.001			3.570
		Subgroup 1: previous use of AI	4 (0)		4							
		Subgroup 2: no previous use of AI	4 (3.5-4.5)		4							
	**S6: ...improve the process of diagnosis**								.002			3.089
		Subgroup 1: previous use of AI	4 (3.5-4.5)		4							
		Subgroup 2: no previous use of AI	4 (3-4)		4							
	**S7: ...improve the process of therapy selection**								<.001			3.865
		Subgroup 1: previous use of AI	4 (0-0)		4							
		Subgroup 2: no previous use of AI	4 (3-4)		4							
	**S8: ...negatively affect the doctor-patient relationship**								.18			1.328
		Subgroup 1: previous use of AI	3 (2-4)		2							
		Subgroup 2: no previous use of AI	3 (2-4)		3							
	**S9: ...lead to a dehumanization of medicine**								.11			1.610
		Subgroup 1: previous use of AI	3 (2-4)		2							
		Subgroup 2: no previous use of AI	3 (2-4)		3							
	**S10: ...negatively affect patient autonomy**								.05			2.040
		Subgroup 1: previous use of AI	3 (2-3)		2							
		Subgroup 2: no previous use of AI	3 (2-4)		3							
	**S11: ...negatively affect the autonomy of medical staff**								.16			1.415
		Subgroup 1: previous use of AI	3 (2-4)		2							
		Subgroup 2: no previous use of AI	3 (2-4)		3							
	**S12: ...bring new ethical challenges**								.02			2.302
		Subgroup 1: previous use of AI	5 (4-5)		5							
		Subgroup 2: no previous use of AI	4 (3-4)		4							

### Part 4: Teaching AI in Medical Education

When asked about their agreement on whether AI teaching should be incorporated into medical education (S13), 74.9% (385/487) of the respondents agreed or strongly agreed. A statistically significant difference was identified between those with and without prior use of AI-based (chat) applications (*P*=.02). The mean (5), mode (5), and *z* score (2.381) suggest higher agreement within the group that previously used AI-based applications. In contrast, there was an overall disagreement (88%) with the assertion that AI instruction in medical education is currently sufficient (S14), with no statistically significant difference between the 2 groups. No significant statistical differences were observed for statements S15-S19. There was an overall agreement that the teaching of AI should include practical content (S15; 417/487, 86%), be based on case studies and application scenarios in medicine (S16; 342/487, 70.3%), be an important prerequisite for medical practice (S17; 314/487, 64.9%), be available to medical staff even after graduation (S18; 376/487, 77.3%), and be updated regularly to reflect advances in AI technology (S19; 407/487, 83.6%). There was a significant measurable difference in the S20 (*P*=.002) between the 2 groups. The *z* score indicates a stronger agreement with the statement “AI instruction is of interest to me” among the group of medical students who previously used AI-based (chat) applications (*z* score: 3.173). The statistical analysis is presented in Table 5, and an overview of the perceptions of the participants regarding the teaching of AI in medicine can be found in Table S2 in Multimedia Appendix 1.

**Table 5:** Statistical analysis of the perceptions of medical students regarding the teaching of artificial intelligence (AI)–based (chat) applications such as ChatGPT (OpenAI), Bard (Google), Bing Chat (Microsoft Inc), and Jasper Chat (Jasper AI, Inc) in medical education (n=487).

Statement and subgroup				Scores, median (IQR)		Scores, mode			*P* value			*Z* score
The teaching of AI...												
	**S13: ...should be part of medical education**							.02			2.381	
		Subgroup 1: previous use of AI	5 (4-5)		5							
		Subgroup 2: no previous use of AI	4 (3-4)		4							
	**S14: ...in medical education is adequate**							.90			0.128	
		Subgroup 1: previous use of AI	2 (1-2)		1							
		Subgroup 2: no previous use of AI	2 (1-2)		1							
	**S15: ...should include practical content (e.g., exercises to apply AI) in addition to theoretical aspects**							.18			−2.358	
		Subgroup 1: previous use of AI	4 (3.5-4.5)		4							
		Subgroup 2: no previous use of AI	4 (0)		4							
	**S16: ...should be based on case studies and application scenarios of AI in medicine**							.53			−0.625	
		Subgroup 1: previous use of AI	4 (3-5)		4							
		Subgroup 2: no previous use of AI	4 (3.5-4.5)		4							
	**S17: ...is an important prerequisite for medical practice**							.16			1.417	
		Subgroup 1: previous use of AI	4 (3.5-4.5)		4							
		Subgroup 2: no previous use of AI	4 (3-4)		4							
	**S18: ...should be available for medical staff even after graduation**							.13			−1.527	
		Subgroup 1: previous use of AI	4 (3.5-4.5)		4							
		Subgroup 2: no previous use of AI	4 (3-5)		4							
	**S19: ...should be updated regularly to reflect advances in AI technology**							.34			−2.121	
		Subgroup 1: previous use of AI	4 (3-4)		4							
		Subgroup 2: no previous use of AI	4 (3-4)		4							
	**S20: ...is of interest to me**							.002			3.173	
		Subgroup 1: previous use of AI	4 (4-5)		4							
		Subgroup 2: no previous use of AI	4 (0)		4							

### Part 5: Teaching AI Ethics in Medical Education

In the survey, 74.9% (385/487) of medical students agreed or strongly agreed that teaching AI ethics should be included in medical education (S21). However, only 4.9% (24/487) agreed that the current instruction on AI ethics in medical education is adequate (S22). For statements S23 to S27, the vast majority of medical students generally agreed (“agree” or “strongly agree”) that the teaching of AI ethics should be based on case studies and application scenarios of AI in medicine (S23; 412/487, 85%), contribute to raising awareness of ethical issues in medical practice (S24; 343/487, 70.6%), is an important prerequisite for medical practice (S25; 354/487, 72.8%), should be available for medical staff even after graduation (S26; 370/487, 75.9%), and should be taught by experts from various fields (eg, medicine, computer science, and philosophy) to ensure a multidisciplinary perspective on AI ethics (S27; 416/487, 85.2%). No statistically significant differences were observed for statements S21 to S27 between the 2 groups (those with previous use of AI-based [chat] applications and those without). Despite the *z* score of 1.782 being below the typical threshold of 1.96 for a 2-tailed test, the statement “the teaching of AI ethics is of interest to me” (S28) showed a statistically significant difference (*P*=.005). This indicates that even though the deviation from the mean agreement level is not as strong as typically expected for significance, those who had previously used AI-based (chat) applications demonstrated a notably higher level of interest in AI ethics teaching than those who had not. The statistical analysis for part 5 of the survey is shown in Table 6, and the distribution of answers is presented in Table S3 in Multimedia Appendix 1.

**Table 6:** Statistical analysis of the perceptions of medical students regarding the teaching of artificial intelligence (AI)–based (chat) applications such as ChatGPT (OpenAI), Bard (Google), Bing Chat (Microsoft Inc), and Jasper Chat (Jasper AI, Inc) ethics in medical education (n=487).

Statement and subgroup				Scores, median (IQR)		Scores, mode			*P* value			*Z* score
The teaching of AI...												
	**S13: ...should be part of medical education**							.37			−0.903	
		Subgroup 1: previous use of AI	5 (4-5)		5							
		Subgroup 2: no previous use of AI	4 (4-5)		5							
	**S14: ...in medical education is adequate**							.21			−1.263	
		Subgroup 1: previous use of AI	2 (2-3)		2							
		Subgroup 2: no previous use of AI	2 (1-2)		1							
	**S15: ...should include practical content (e.g., exercises to apply AI) in addition to theoretical aspects**							.80			−0.254	
		Subgroup 1: previous use of AI	4 (0)		4							
		Subgroup 2: no previous use of AI	4 (0)		4							
	**S16: ...should be based on case studies and application scenarios of AI in medicine**							.48			−0.707	
		Subgroup 1: previous use of AI	4 (3-4)		4							
		Subgroup 2: no previous use of AI	4 (2.5-4.5)		4							
	**S17: ...is an important prerequisite for medical practice**							.90			0.118	
		Subgroup 1: previous use of AI	4 (3-4)		4							
		Subgroup 2: no previous use of AI	4 (2-4)		4							
	**S18: ...should be available for medical staff even after graduation**							.17			−1.359	
		Subgroup 1: previous use of AI	4 (3-4)		4							
		Subgroup 2: no previous use of AI	4 (2-4)		4							
	**S19: ...should be updated regularly to reflect advances in AI technology**							.17			−1.381	
		Subgroup 1: previous use of AI	4 (3-4)		4							
		Subgroup 2: no previous use of AI	4 (3-4)		4							
	**S20: ...is of interest to me**							.005			1.782	
		Subgroup 1: previous use of AI	4 (3-4)		4							
		Subgroup 2: no previous use of AI	4 (0)		4							

### Part 6: AI Ethics Teaching Content

In analyzing the perceptions of medical students with and without prior exposure to AI chat applications regarding AI ethics content, all 8 proposed topics were deemed highly relevant (“quite relevant” and “very relevant”) by the respondents: TC1: 418/487, 85.9%; TC2: 408/487, 83.8%; TC3: 384/487, 78.9%; TC4: 415/487, 85.2%; TC5: 423/487, 86.2%; TC6: 407/487, 83.6%; TC7: 402/487, 82.5%; and TC8: 448/487, 92.3%). No statistically significant difference was observed between the responses of both groups, except for TC1 (informed consent; *P*=.04). The *z* score suggests that medical students who had previously used AI-based (chat) applications perceived informed consent to be more relevant than those who had not (*z* score: 2.018). The statistical results of this section are shown in Table 7, with an overview of the statements on the relevance of AI ethics teaching content provided in Table S4 in Multimedia Appendix 1.

**Table 7:** Statistical analysis of the relevance of artificial intelligence (AI)–based (chat) applications such as ChatGPT (OpenAI), Bard (Google), Bing Chat (Microsoft Inc), and Jasper Chat (Jasper AI, Inc) ethics teaching contents according to the participating medical students (n=487).

AI ethics teaching content and subgroup		Scores, median (IQR)	Scores, mode	*P* value	*Z* score
**TC1: informed consent**				.04	2.018
	Subgroup 1: previous use of AI	4 (4-5)	5		
	Subgroup 2: no previous use of AI	4 (3-4)	4		
**TC2: bias**				.22	−1.215
	Subgroup 1: previous use of AI	4 (4-5)	5		
	Subgroup 2: no previous use of AI	4 (3-4)	4		
**TC3: data privacy**				.78	0.283
	Subgroup 1: previous use of AI	4 (4-5)	5		
	Subgroup 2: no previous use of AI	4 (4-5)	5		
**TC4: explainability**				.36	−0.911
	Subgroup 1: previous use of AI	4 (4-5)	5		
	Subgroup 2: no previous use of AI	4 (3.5-4.5)	4		
**TC5: safety**				.57	0.565
	Subgroup 1: previous use of AI	5 (4-5)	5		
	Subgroup 2: no previous use of AI	5 (4-5)	5		
**TC6: fairness**				.96	−0.048
	Subgroup 1: previous use of AI	4 (4-5)	5		
	Subgroup 2: no previous use of AI	4 (4-5)	5		
**TC7: autonomy**				.11	1.594
	Subgroup 1: previous use of AI	4 (4-5)	5		
	Subgroup 2: no previous use of AI	4 (4-5)	5		
**TC8: responsibility**				.22	−1.215
	Subgroup 1: previous use of AI	5 (4-5)	5		
	Subgroup 2: no previous use of AI	5 (4-5)	5		

### Additional Analysis of the Collected Data

To analyze whether there is a difference in education regarding AI and AI ethics among Germany, Austria, and Switzerland, we conducted an additional evaluation of the collected data. For this supplementary analysis, we analyzed the responses to Q2: “Have you already received education in the field of artificial intelligence within your regular medical studies? (eg, as part of medical statistics or informatics),” and Q4: “Have you already received education in the field of AI ethics within your regular medical studies? (eg, as part of the History, Ethics, and Theory of Medicine course).” Using the chi-square test of independence, we sought to determine whether the distribution of answers varied significantly among these countries. In the comparison between the 3 countries concerning education in the field of AI, the chi-square test of independence indicated no significant difference in the distribution of the responses. Of the 487 respondents, only 26 (5.3%) indicated that they had previously received AI education. The test yielded a result of *χ*^2^_2_(N=487)=0.1 (*P*=.33). Similarly, regarding education in the field of AI ethics, the distribution of responses among the countries was not significantly different. Of the 487 respondents, only 21 (4.3%) indicated that they had received education on AI ethics. The test yielded a result of *χ*^2^_2_(N=487)=0.3 (*P*=19).

### Stage of Study

To account for potential confounders, such as the stage of the study, further analyses were performed on the data set. Recognizing the possible overlaps and similarities in experiences and perspectives across the different stages, the original 6 stages of the study were further consolidated. The stages “preclinical” and “bachelor” were summarized into the “preclinical stage (PCS).” Similarly, the “clinical” and “master” stages were combined into the “clinical stage.” Finally, the “practical year” and “elective year” stages were grouped together to form the “clinical practical stage (CPS).” With these redefined categories, the chi-square test of independence was used to analyze whether there were significant variations in perceptions and responses across the 3 consolidated stages.

Focusing on the potential impact of AI in medicine, a significant difference was observed in the statement, “the use of AI in medicine will influence the choice of my specialization” (S3). CPS participants were notably more influenced than those in the PCS (*P*=.004). However, no difference was evident between the PCS and CS participants. Most other statements concerning AI’s impact on medicine (S1-2; S4-12) did not demonstrate statistical significance. Similarly, no significant difference was found for statements related to AI teaching (S13-20) across the study stages (PCS, CS, and CPS). When considering the teaching of AI ethics, differences were evident in the belief that AI ethics should be integrated into medical education (S21; *P*=.003) and that the current teaching of AI ethics is adequate (S22; *P*=.02). Upon further analysis, CS participants showed stronger agreement than PCS participants, with no difference when compared with CPS participants. Finally, for the specific content of AI ethics teaching, none of the statements reflected significant statistical variation across the study stages. An overview of the statistical differences is provided in Tables S5-S8 in Multimedia Appendix 1.

### Ethics Education Background

To explore the potential impact of prior ethics education on survey outcomes, particularly in parts 3 to 6, we compared 2 distinct groups: those with prior ethics education and those without. On the use of AI in medicine, one statistical difference could be determined for the statement that “...negatively affect the autonomy of medical staff’ (S11, *P*=.002). The *z* score suggested a stronger level of agreement with the statement in the group that had received prior ethics education (*z* score: 2.876). For the other statements of the third part of the survey (S1-10; S12), no statistical difference could be determined. No statistical difference could be determined for the fourth part of the survey on AI teaching (S13-20). Regarding the teaching of AI ethics, statistical differences could be determined for 2 statements (S21, *P*=.004; S22, *P*=.03). For the statement that the teaching of AI ethics should be part of medical education, the *z* score indicated a higher level of agreement in the group that had received prior ethics education. Similarly, a higher level of disagreement was indicated by the group with prior ethics education for the statement that the teaching of AI ethics in medical education is adequate (*z* score: −3.011). There was no statistically significant difference in the AI ethics teaching content between the groups. A detailed statistical analysis can be found in Tables S9-S12 in Multimedia Appendix 1.

## Discussion

This discussion aims to comprehensively analyze the findings regarding medical students’ perceptions of AI in medicine and the role of AI and AI ethics in their medical education, depending on their use of AI-based (chat) applications such as ChatGPT.

### The Use of AI-Based (Chat) Application Among the Surveyed Medical Students

The discrepancy between students’ personal AI experiences and formal medical education highlights the gap in integrating AI into curricula, reflecting the need for educational progress in line with technological advancement. A considerable 38.8% of the respondents reported prior use of AI-based (chat) applications, such as ChatGPT, Bard, Bing Chat, or Jasper Chat, which was slightly below the percentage received from pretesting and used for sample size calculation (5/11, 45%). The results concerning the reported use of AI-based (chat) applications must be evaluated in the context of the timing of the data collection. ChatGPT, for instance, became freely available to the public on November 30, 2022, making it accessible for only approximately 8 months at the time of data collection [27]. In addition, Bing Chat was not broadly accessible until May 2023, further constraining its availability before the survey [28]. It is noteworthy that academic literature on the use of AI-based (chat) applications such as ChatGPT among medical students is still limited. A study conducted with health students found that only 11.3% (55/458) of respondents reported using the ChatGPT, a rate considerably lower than the findings of this study [29].

A more detailed evaluation of the percentage of medical students using AI-based (chat) applications is necessary given that many might use AI unknowingly. This is not restricted to clinical AI tools, such as clinical decision support systems but extends to search engines and other tools. For example, the search engine Bing offers AI-driven content with search results, irrespective of whether the Bing chat is specifically used. Moreover, a study conducted with students from various specialties in Germany revealed that 12.3% (779/6311) of its participants used “DeepL” (DeepL SE), an AI-based translation tool, in which the use of AI might not be immediately evident [30]. Therefore, when considering other AI tools and applications, the actual percentage of medical students using them may be significantly higher than the 38.8% reported in this study. Recognizing this potential underestimation of AI use highlights the importance of expanding AI literacy and awareness in medical education to ensure that future health care professionals are adequately prepared for the integration of AI in medicine. This reinforces the need for proactive measures in curriculum design to include not only the direct use of AI tools but also an understanding of their indirect implications in various medical and research contexts.

### AI Education

Despite the significant engagement of students with AI-based applications, such as ChatGPT, only a small fraction (26/487, 5.3%) reported formal AI education within their medical curriculum. This discrepancy highlights the critical gap between experiential learning and structured academic guidance regarding AI. Interestingly, AI education outside the formal curriculum was more prevalent (51/487, 10.5%), which could imply a proactive approach to learning about AI technologies. Furthermore, this could be attributed to the availability of AI-based applications, such as ChatGPT, and increasing opportunities for education on AI in the medical context, as well as AI-based (chat) applications that are knowledgeable in the field of medicine [7,31-33]. Among the users of AI applications, 73% applied these tools in medical contexts, primarily for querying medical knowledge. This use pattern presents both opportunities for accessible knowledge and risks associated with reliance on uncertified AI sources and a lack of certification as medical devices. The lack of education in the field of AI as part of medical education has been highlighted not only in German-speaking countries [34] but also internationally [21,22].

The results imply a substantial dichotomy between the lack of formal education and optimism toward AI, as the use of AI in medicine was positively perceived (71.1% of respondents), despite the absence of formal education (94.7% of respondents). Given the lack of education, this warrants caution as there might be an overly optimistic view of its potential benefits, overlooking potentially significant limitations and ethical implications [35]. The need for the integration of AI into medical curricula is not only supported by existing studies highlighting low AI literacy among medical students [34,36] but also by the results of this study, with 88% of all medical students perceiving that their current AI education within their medical education is insufficient. This dissatisfaction underscores the need for medical curricula to evolve in tandem with technological advancements. However, it is crucial to ensure that these curricular changes are developed thoughtfully and comprehensively to avoid superficial or overly optimistic portrayals of AI’s role of AI in medicine [34]. The findings of this study, indicating a significant gap in AI education within medical curricula, align with the initial insights gathered regarding students’ use of AI applications. Furthermore, the results align with the objective of understanding how medical students from Germany, Austria, and Switzerland perceive the application of AI in medical practice and its integration into medical education. This disparity between the practical use of AI applications and lack of AI educational opportunities in the curriculum underlines the emerging need for educational reform.

### AI Ethics Education

The perceived insufficiency of the current medical education extends to AI ethics. Remarkably, 95.3% of participants acknowledged the new ethical challenges posed by AI in medicine, which resonates with preexisting research [15]. Notably, those who used AI-based (chat) applications, such as ChatGPT, agreed more strongly with this view, suggesting that practical use enhances awareness of these ethical issues. In addition, 74.9% (385/487) of respondents recognized the necessity of integrating AI ethics into medical curricula, aligning with recent academic discourse [37-39]. However, only a small percentage (4.3%) reported formal AI ethics education, highlighting a significant deficit in the current curriculum. Medical students perceived all 8 proposed ethical AI topics as highly relevant, which were recommended as potential teaching content for AI ethics in the current literature [37-39]. Statistical differences were observed for “informed consent” among those with prior AI application use. This indicates that engagement with AI technology may deepen understanding of its ethical dimensions, reinforcing the need for comprehensive AI ethics instruction in medical education. The clear demand for AI ethics education reflects a broader educational need, where medical students should not just be prepared for the technicalities of AI but also for the nuanced ethical considerations introduced by the technology.

Although this study underscores the need for both AI and AI ethics education in medical curricula, it is also important to critically assess the current absence of AI-centric content. Rapid technological advancements in AI with the recent public availability of AI tools, such as ChatGPT, may contribute to the current lack of associated teaching content. Given the complex regulatory requirements required to use AI-based technologies in clinical practice, the use of AI in medicine is currently not widespread [40]. In addition, the requirement for time-consuming and complex reaccreditation processes for curricular development and revision may further delay the introduction of AI-related teaching content [41]. Moreover, the lack of widespread use of AI-based applications in medicine and clinical practice likely contributes to the current lack of adequate teaching content on AI and ethics. The overwhelming perception of AI’s potential and its ethical implications it brings forth, as evidenced by this study, underscores the need for educational institutions to respond proactively. Balancing the speed of technological advancements in the field of AI with thoughtful and comprehensive curricular integration is likely to be a crucial challenge in medical education in the coming years.

### Additional Analysis of the Collected Data

In the additional data analysis, the subsequent examination revealed that perceptions of AI and AI ethics among medical students were not significantly influenced by their country of study. This uniformity across Germany, Austria, and Switzerland suggests consistency in deficiencies in AI and AI ethics education regardless of regional curricular variations. As the findings could be attributed to the limited number of medical students indicating prior education in AI (26/487, 5.3%) and AI ethics (21/487, 4.3%), additional research is warranted. Despite their different educational systems, the observed uniformity in AI and AI ethics education across the 3 countries implies a broader challenge for medical education. The consistency of educational deficiencies, irrespective of regional curricular variations, indicates the widespread need to reform AI teaching in medical curricula. This aligns with the overarching findings of our study, which suggest a universal gap in AI competencies among medical students.

Further analysis of the study stage revealed that students in advanced stages, such as CPS, showed increased awareness of the potential impact of AI on their specialization choices, implying a growing realization of AI’s role as they progress in their studies. However, the lack of significant differences in most other AI-related statements could also imply a generalized consensus or a lack of adequate exposure and understanding across all study stages. As an advancement in the study stages could be linked to statistically significant results on statements regarding the need to teach AI ethics, this could be attributable to prior ethics education, which is usually taught during the PCSs.

The impact of ethics education on perceptions of AI’s role in medicine is particularly notable. Students with such an education showed increased awareness of the ethical challenges posed by AI, especially regarding its potential negative impact on medical staff autonomy (S11). This could underscore the importance of ethics education in understanding the potentially wide-reaching challenges of AI in medicine for ethically important subjects such as autonomy; however, no statistically significant difference for the preceding statement on autonomy “the use of AI in medicine will negatively affect patient autonomy” (S10) could be observed. This could imply that prior ethics education, including teaching autonomy in a medical context, might lead to a more nuanced understanding of the subject and potential implications of AI. The results of the analysis reinforce the need for ongoing ethics education, not just as a separate entity, but also interwoven with AI-related topics, to enhance students’ comprehensive understanding of the ethical implications of AI in medicine. The significant influence of prior ethics education on shaping students’ perceptions of the role of AI in medicine emphasizes the interaction between ethical training and technological awareness. The nuanced understanding of the ethical implications of AI among students who have received ethics education underscores the importance of such training in developing critical thinking about the impact of AI in health care. Integrating ethics education with AI teaching content could foster a more holistic approach, preparing students not only for the technological aspects of AI but also for its ethical and societal implications [37].

### Limitations

Despite the strengths of this study, some limitations must be acknowledged. First, our web-based survey could introduce selection bias, as tech-savvy students may be more likely to participate. Second, the survey measured students’ perceptions rather than their actual competencies in AI and ethics. In addition, although estimated, the response rate was suboptimal, which may limit the generalizability of our findings. Geographically, our sample was limited to German-speaking countries, making the translation of these results to other countries with different health care systems and medical educational frameworks difficult. Cultural attitudes toward AI could also vary, possibly influencing students’ perceptions of and engagement with AI. Our study is essentially a snapshot of a rapidly evolving field; hence, our findings may not reflect attitudes and competencies, as they evolve with advancements in AI technology. In our analysis, we observed statistically significant differences based on prior ethics education and study stage. However, although the additional analysis of the data did not show a direct overlap with significant findings between the main and supplementary evaluations, additional tests are needed to determine whether these factors acted as confounders in our main data analysis. Although this study considered specific potential confounders, it is worth noting that other confounding variables may exist and were not analyzed in this study. Finally, owing to the self-reported nature of the data, the responses might be subject to recall bias, misunderstanding of questions, or social desirability bias. Although our findings provide valuable insights into the state of AI education in German-speaking medical schools, broader multinational studies would offer a more comprehensive understanding.

### Conclusions

This study provides a valuable understanding of the perceptions and experiences of medical students in Germany, Austria, and Switzerland regarding the application of AI in medicine, and its role in medical education. Our findings clearly indicate a discrepancy between students’ interactions with AI-based chat applications such as ChatGPT and the representation of AI in their formal education. Despite a significant number of students interacting with AI technology, notably AI-based chat applications, only a fraction have received any formal AI education, revealing a substantial gap in the current medical curricula. This highlights the necessity of the evolution of medical curricula to incorporate AI and AI ethics education, ensuring that future medical professionals are adequately equipped to navigate the challenges and opportunities presented by AI in medicine.

Furthermore, our findings indicate that practical engagement with AI technology can contribute to an increased awareness of ethical implications, reinforcing the importance of including hands-on AI experiences in medical education. It is evident that the rapid advancement and application of AI in medicine demands parallel evolution in medical education. Thoughtful and comprehensive curricular changes are required to provide a balanced understanding of the potential benefits, limitations, and ethical implications of AI. The integration of AI and AI ethics into medical education is an urgent necessity, not only to enhance students’ AI literacy but also to ensure the responsible and effective use of AI in future medical practice demands.

## Appendix

Multimedia Appendix 1 Comprehensive statistical analysis and evaluation of confounding factors regarding medical students’ perceptions of artificial intelligence’s role in medicine and medical education.

## Abbreviations

AI: artificial intelligence
CPS: clinical practical stage
CS: clinical stage
PCS: preclinical stage
