# Supplementary material for: Artificial Intelligence in Medicine: Cross-Sectional Study Among Medical Students on Application, Education, and Ethical Aspects
Source: JMIR Med Educ. 2024 Jan 5;10:e51247. doi: 10.2196/51247 (PMC10799276; doi:10.2196/51247)
Supplement: Multimedia Appendix 1 [file mededu_v10i1e51247_app1.pdf]

Multimedia Appendix 1. Comprehensive statistical analysis and confounding factors evaluation in medical students' perceptions of artificial intelligence's role in medicine and medical education.

Table S1: Perceptions of the surveyed medical students on the use of AI in medicine (n = 487)

**Statement**	**n (%)**				
**The use of artificial intelligence in medicine will...**					
	**I strongly disagree**	**I disagree**	**Undecided**	**I agree**	**I strongly agree**
S1: ...positively change medicine	5 (1.0)	55 (11.3)	78 (16.0)	239 (49.1)	110 (22.6)
S2: ...find useful applications in medicine	15 (3.1	54 (11.1)	68 (14.0)	221 (45.5)	129 (26.6)
S3: ...influence the choice of my medical specialization	114 (23.4)	126 (25.9)	77 (15.8)	149 (30.6)	21 (4.3)
S4: ...reduce the number of jobs for medical staff	82 (16.8)	135 (27.7)	103 (21.2)	143 (29.4)	24 (4.9)
S5: ...improve the quality of patient care	5 (1.0)	19 (3.9)	93 (19.2)	287 (53.9)	83 (17.1)
S6: ...improve the process of diagnosis	3 (0.6)	18 (3.7)	79 (16.2)	288 (59.2)	99 (20.4)
S7: ...improve the process of therapy selection	3 (0.6)	23 (4.7)	82 (16.9)	293 (60.3	86 (17.6)
S8: ...negatively affect the doctor-patient relationship	39 (8.0)	150 (30.8)	143 (29.3)	139 (28.5)	16 (3.3)
S9: ...lead to a dehumanization of medicine	51 (10.5)	148 (30.4)	144 (29.6)	127 (26.1)	17 (3.5)
S10: ...negatively affect patient autonomy	45 (9.3)	159 (32.9)	158 (32.7)	113 (23.4)	12 (2.5)
S11: ...negatively affect the autonomy of medical staff	40 (8.2)	141 (29.1)	152 (31.3)	139 (28.7)	15 (3.1)
S12: ...bring new ethical challenges	3 (0.6)	3 (0.6)	17 (3.5)	229 (47.0)	235 (48.3)

Table S2: Perceptions of the surveyed medical students on the teaching of AI in medicine (n = 487)

**Statement**	**n (%)**				
**The teaching of AI…**					
	**I strongly disagree**	**I disagree**	**Undecided**	**I agree**	**I strongly agree**
S13: ...should be part of medical education.	25 (5.1)	32 (6.6)	65 (13.6)	174 (35.7)	191 (39.2)
S14: ...in medical education is adequate	228 (47.1)	198 (40.9)	50 (10.3)	8 (1.7)	3 (0.6)
S15: ...should include practical content (e.g., exercises to apply AI) in addition to theoretical aspects	3 (0.6)	9 (1.9)	58 (12.0)	292 (60.2)	125 (25.8)
S16: ...should be based on case studies and application scenarios of AI in medicine	2 (0.4)	8 (1.6)	135 (27.8)	233 (47.9)	109 (22.4)
S17: ...is an important prerequisite for medical practice	17 (3.5)	47 (9.7)	109 (22.5)	220 (45.5)	94 (19.4)
S18: ... should be available for medical staff even after graduation	2 (0.4)	5 (1.0)	104 (21.4)	244 (50.2)	132 (27.1)
S19: ... should be updated regularly to reflect advances in AI technology	2 (0.4)	5 (1.0)	73 (15.0)	259 (53.2)	148 (30.4)
S20: ...is of interest to me	6 (1.2)	26 (5.3)	51 (10.5)	320 (65.8)	84 (17.3)

Table S3: Perceptions of the surveyed medical students on the teaching of AI ethics in medicine (n = 487)

**Statement**	**n (%)**				
**The teaching of AI ethics…**					
	**I strongly disagree**	**I disagree**	**Undecided**	**I agree**	**I strongly agree**
S21: ...should be part of medical education.	27 (5.5)	42 (8.6)	53 (10.9)	173 (35.5)	192 (39.4)
S22: ...in medical education is adequate	220 (45.2)	191 (39.3)	52 (10.7)	18 (3.7)	6 (1.2)
S23: ...should be based on case studies and application scenarios of AI in medicine	5 (1.0)	9 (1.8)	61 (12.6)	303 (62.5)	109 (22.5)
S24: ...contributes to raising awareness for ethical issues in medical practice.	4 (0.8)	14 (2.9)	126 (26.0)	225 (46.4)	118 (24.2)
S25: ...is an important prerequisite for medical practice	17 (3.5)	29 (6.0)	87 (17.9)	245 (50.4)	109 (22.4)
S26: ...should be available for medical staff even after graduation	3 (0.6)	10 (2.1)	104 (21.4)	236 (48.6)	134 (27.3)
S27: ...should be taught by experts from various fields (e.g., medicine, computer science, philosophy) to ensure a multidisciplinary perspective on AI ethics	4 (0.8)	4 (0.8)	65 (13.4)	272 (56.0)	142 (29.2)
S28: ...is of interest to me	7 (1.4)	22 (4.5)	55 (11.2)	315 (64.9)	88 (18.1)

Table S4: Relevance of AI ethics teaching contents according to the participating medical students (n = 487)

**AI ethics teaching content**	**n (%)**				
	**Not relevant**	**Slightly relevant**	**Moderately relevant**	**Quite relevant**	**Very relevant**
**TC1: Informed Consent**<br><br>*Given the complexity of AI, it is questionable whether doctors will be able to understand the technology itself in the clinical context to such an extent that conveying relevant knowledge to patients will be possible, enabling them to make an informed decision.*	3 (0.6)	15 (3.1)	51 (10.5)	206 (42.4)	212 (43.5)
**TC2: Bias**<br><br>*The use of AI in medicine can lead to discrimination if the data used for training or programming the AI lack representativeness.*	5 (1.0)	5 (1.0)	69 (14.2)	233 (47.8)	175 (36.0)
**TC3: Data Privacy**<br><br>*As the use of AI in medicine involves highly sensitive patient data, security gaps or data misuse can have far-reaching consequences.*	3 (0.6)	25 (5.1)	75 (15.4)	149 (30.6)	235 (48.3)
**TC4: Explainability**<br><br>*Decisions made by AI-based applications cannot always be traced by the users due to the technical structure and complexity.*	3 (0.6)	15 (3.1)	54 (11.1)	210 (43.1)	205 (42.1)

**TC5: Safety**<br><br>*If AI-based applications are used for medical purposes, such as in diagnosis or treatment decision-making, faulty programming could potentially lead to significant hazards for patients.*	4 (0.8)	11 (2.3)	49 (10.1)	164 (33.7)	259 (53.1)
**TC6: Fairness**<br><br>*In addition to fairness in terms of equal treatment by the AI-based applications used (e.g., risk of bias and discrimination), access to the technology itself also plays a crucial role.*	4 (0.8)	17 (3.5)	59 (12.1)	182 (37.4)	225 (46.2)
**TC7: Autonomy**<br><br>*The use of AI in medicine can limit the autonomy of patients (e.g., regarding the use of AI in their own treatment) and doctors (e.g., in the freedom to decide on treatment recommendations).*	6 (1.2)	14 (2.9)	65 (13.4)	171 (35.1)	231 (47.4)
**TC8: Responsibility**<br><br>*In the event of treatment errors when using AI in medicine, the question of liability and responsibility on the part of the users arises.*	0 (0.0)	6 (1.2)	33 (6.8)	157 (32.3)	291 (59.7)

Stage of Study

Table S5: Perceptions of the surveyed medical students on the use of AI in medicine across study stages (n = 487)

Statement	*P* value (Chi-Square)	Post-Hoc Analysis (Adjusted Residuals)
**The use of artificial intelligence in medicine will...**		
S1: ...positively change medicine	.21	
S2: ...find useful applications in medicine	.36	
S3: ...influence the choice of my medical specialization	**.004**	CPS > PCS; No difference between PCS and CS
S4: ...reduce the number of jobs for medical staff	.45	
S5: ...improve the quality of patient care	.41	
S6: ...improve the process of diagnosis	.24	
S7: ...improve the process of therapy selection	.17	
S8: ...negatively affect the doctor-patient relationship	.23	
S9: ...lead to a dehumanization of medicine	.35	
S10: ...negatively affect patient autonomy	.58	
S11: ...negatively affect the autonomy of medical staff	.37	
S12: ...bring new ethical challenges	.78	

Table S6: Perceptions of the surveyed medical students on the teaching of AI in medicine across study stages (n = 487)

Statement	*P* value (Chi-Square)	Post-Hoc Analysis (Adjusted Residuals)
**The teaching of AI…**		
S13: ...should be part of medical education.	.73	
S14: ...in medical education is adequate	.68	
S15: ...should include practical content (e.g., exercises to apply AI) in addition to theoretical aspects	.52	
S16: ...should be based on case studies and application scenarios of AI in medicine	.45	
S17: ...is an important prerequisite for medical practice	.39	
S18: ... should be available for medical staff even after graduation	.38	
S19: ... should be updated regularly to reflect advances in AI technology	.13	
S20: ...is of interest to me	.22	

Table S7: Perceptions of the surveyed medical students on the teaching of AI ethics in medicine across study stages (n = 487)

Statement	*P* value (Chi-Square)	Post-Hoc Analysis (Adjusted Residuals)
The teaching of AI ethics…		
S21: ...should be part of medical education.	**.003**	CS > PCS; No difference between CS and CPS
S22: ...in medical education is adequate	**.02**	CS > PCS; No difference between CS and CPS
S23: ...should be based on case studies and application scenarios of AI in medicine	.36	
S24: ...contributes to raising awareness for ethical issues in medical practice.	.41	
S25: ...is an important prerequisite for medical practice	.53	
S26: ...should be available for medical staff even after graduation	.48	
S27: ...should be taught by experts from various fields (e.g., medicine, computer science, philosophy) to ensure a multidisciplinary perspective on AI ethics	.27	
S28: ...is of interest to me	.58	

Table S8: Relevance of AI ethics teaching contents according to the participating medical students across study stages (n = 487)

**AI ethics teaching content**	***P* value (Chi-Square)**	**Post-Hoc Analysis (Adjusted Residuals)**
TC1: Informed Consent	.31	
TC2: Bias	.42	
TC3: Data Privacy	.17	
TC4: Explainability	.23	
TC5: Safety	.56	
TC6: Fairness	.61	
TC7: Autonomy	.11	
TC8: Responsibility	.23	

Ethics Education Background

Table S9: Perceptions of the surveyed medical students on the use of AI in medicine based on prior ethics education (n = 487)

**Statement**	***P* value**	**Z-score**
The use of artificial intelligence in medicine will...		
S1: ...positively change medicine	.12	0.883
S2: ...find useful applications in medicine	.43	-1.142
S3: ...influence the choice of my medical specialization	.16	-1.479
S4: ...reduce the number of jobs for medical staff	.29	1.385
S5: ...improve the quality of patient care	.36	-1.268
S6: ...improve the process of diagnosis	.51	1.151
S7: ...improve the process of therapy selection	.63	-0.931
S8: ...negatively affect the doctor-patient relationship	.49	1.674
S9: ...lead to a dehumanization of medicine	.52	1.572
S10: ...negatively affect patient autonomy	.58	-1.332
S11: ...negatively affect the autonomy of medical staff	**.002**	2.876
S12: ...bring new ethical challenges	.11	1.549

Table S10: Perceptions of the surveyed medical students on the teaching of AI in medicine based on prior ethics education (n = 487)

**Statement**	***P* value**	**Z-score**
**The teaching of AI…**		
S13: ...should be part of medical education.	.37	-1.432
S14: ...in medical education is adequate	.48	-1.563

S15: ...should include practical content (e.g., exercises to apply AI) in addition to theoretical aspects	.13	-1.333
S16: ...should be based on case studies and application scenarios of AI in medicine	.76	0.972
S17: ...is an important prerequisite for medical practice	.52	-0.914
S18: ... should be available for medical staff even after graduation	.29	1.375
S19: ... should be updated regularly to reflect advances in AI technology	.21	1.242
S20: ...is of interest to me	.14	-1.178

Table S11: Perceptions of the surveyed medical students on the teaching of AI ethics in medicine based on prior ethics education (n = 487)

**Statement**	***P* value**	**Z-score**
**The teaching of AI ethics…**		
S21: ...should be part of medical education.	**.004**	2.674
S22: ...in medical education is adequate	**.03**	-3.011
S23: ...should be based on case studies and application scenarios of AI in medicine	.22	-1.138
S24: ...contributes to raising awareness for ethical issues in medical practice.	.42	-0.994
S25: ...is an important prerequisite for medical practice	.51	0.897
S26: ...should be available for medical staff even after graduation	.59	-1.321
S27: ...should be taught by experts from various fields (e.g., medicine, computer science, philosophy) to ensure a multidisciplinary perspective on AI ethics	.37	-1.114
S28: ...is of interest to me	.71	1.457

Table S12: Relevance of AI ethics teaching contents according to the participating medical students based on prior ethics education (n = 487)

**AI ethics teaching content**	***P* value**	**Z-score**
TC1: Informed Consent	.41	0.981
TC2: Bias	.23	-0.532
TC3: Data Privacy	.51	-1.561
TC4: Explainability	.62	-1.117
TC5: Safety	.27	0.989

TC6: Fairness	.38	-1.245
TC7: Autonomy	.47	-0.727
TC8: Responsibility	.13	1.336
